# Does fructose play a role in metabolic disorders?

**DOI:** 10.1186/1753-6561-6-S3-O24

**Published:** 2012-06-01

**Authors:** Luc Tappy

**Affiliations:** 1 Department of Physiology and Service of Endocrinology, Diabetes and Metabolism Lausanne University Hospital, Lausanne, Switzerland

## 

There is evidence that high fructose diets can lead to the development of obesity, insulin resistance, and dyslipidemia in rodents model. In humans, however, the role of fructose in the recent increase in the prevalence of metabolic diseases remains much debated. Several epidemiological studies show a positive relationship between consumption of added sugar, fructose, or sweetened beverages on one hand, and metabolic disorders on the other hand, but fail to conclusively prove a causal relationship. Several short term overfeeding studies show that a high fructose diet can, over a short period of time, increase plasma triglyceride concentrations and in intra-hepatocellular lipid concentrations, stimulate hepatic de novo lipogenesis, modestly increase endogenous glucose production, and enhance post-glucose glycemic responses. Whether these studies are relevant to the pathophysiology of the metabolic syndrome is however not known, and several important issues remain however to be addressed. More specifically, whether consumption of fructose causes metabolic disorders when consumedas part of an energy-balanced diet is not known. The interactions between fructose intake and other environmental factors such as other macronutrients or physical activity have also not been addressed. This presentation will summarize the present knowledge regarding the metabolic effects of short term high fructose diets in humans. Novel data will be presented to support that a high fructose diet increases plasma triglycerides independently of energy balance. Finally, the influence of other environmental factors (dietary protein intake, exercise) on the metabolic effects of fructose will be presented.

